# Examining the day-to-day bidirectional associations between physical activity, sedentary behavior, screen time, and sleep health during school days in adolescents

**DOI:** 10.1371/journal.pone.0238721

**Published:** 2020-09-03

**Authors:** Youngdeok Kim, Masataka Umeda, Marc Lochbaum, Robert A. Sloan

**Affiliations:** 1 Department of Kinesiology and Health Sciences, Virginia Commonwealth University, Richmond, Virginia, United States of America; 2 Department of Kinesiology, University of Texas at San Antonio, San Antonio, Texas, United States of America; 3 Department of Kinesiology and Sport Management, Texas Tech University, Lubbock, Texas United States of America; 4 Education Academy, Vytautas Magnus University, Kaunas, Lithuania; 5 Department of Social and Behavioral Medicine, Kagoshima University Graduate Medical School, Kagoshima, Japan; University of Kentucky, UNITED STATES

## Abstract

**Background:**

Adolescence is a vulnerable period for experiencing poor sleep health. Growing studies have demonstrated lifestyle behaviors including physical activity (PA), screen time (SCT), and sedentary behaviors (SED) as the potential factors associated with sleep health in adolescents; yet, the evidence is inconclusive and the directionality of temporal associations across school days are not well understood. This study examined the day-to-day bidirectional associations of lifestyle behaviors with sleep health parameters in adolescents.

**Methods:**

A total of 263 adolescents (58% boys) in 6^th^ - 8^th^ grades wore an accelerometer for 24-hour across the three consecutive school days and completed recording SCT in time-diary and answering sleep quality (SQ) questions for each day. Sleep-wake patterns as well as time spent in moderate- and vigorous-intensity PA (MVPA) and SED were objectively quantified from the wrist-worn accelerometry data across the two segments of the day (during and after school hours). Mixed model analyses were conducted to test bidirectional associations between lifestyle factors and sleep health parameters in each temporal direction across the days. Additionally, indirect associations across the days were tested using an autoregressive cross-lagged model analysis in the framework of path analysis.

**Results:**

MVPA minutes in a day did not predict sleep health parameters that night. The bidirectional associations were partially observed between SED and sleep health, but the significance and direction of the associations largely varied by the time segment of a day as well as types of sleep health parameters. Additionally, greater SCT during the day was associated with lower SQ that night (b = -0.010; *P* = .018), and greater SQ was associated with greater MVPA during school hours (b = 6.45; *P* = .028) and lower SED after school hours (b = -39.85; *P* = .029) the next day. Lastly, there were significant indirect associations of SCT with sleep health parameters across the days indicating multi-day lagged effects of SCT on sleep health the later nights.

**Conclusion:**

This study highlights the importance of lowering SCT for better sleep health in adolescents during school days. Additionally, perceived SQ is shown to be a potential significant predictor promoting healthy behaviors the next day independent of sleep-wake patterns. Further studies are warranted to confirm the observed temporal associations between SCT, SQ, and behavioral outcomes in this vulnerable population.

## Introduction

Sleep is a complex behavior modulating the homeostatic process in the body, which plays an important role in healthy growth and development among adolescents [1]. A large body of literature has demonstrated that poor sleep health (i.e., low sleep quality and quantity) is significantly associated with degraded cognitive and functional performances [2, 3] and increased daytime sleepiness and fatigue [1, 4], which may negatively alter daily behavioral patterns in this population [5, 6]. Unfortunately, adolescents are one of the most vulnerable groups experiencing poor sleep health due to biological changes in circadian rhythm during puberty [7] and sleep deprivation during school days [8]. Recent epidemiological findings highlight a relatively large proportion of US adolescents (68.3%) reporting insufficient sleep quantity [9], with a higher prevalence of poor sleep quality [10].

Of the several factors influencing sleep health, physical activity (PA) has been continuously examined with sleep health among adolescents. It has been hypothesized that PA is associated with better sleep health based on biologically plausible theories of sleep, including energy conservation, body tissue restitution, and temperature down-regulation [11, 12]. There is empirical evidence supporting the beneficial effects of PA on sleep health such as greater likelihoods of reporting sufficient sleep, lower risk of reporting insomnia symptoms, and shorter sleep latency [13–15]. This finding may suggest PA as an effective non-invasive sleep hygiene behavior in adolescents. However, not all studies have reported positive associations. For instance, a large cross-sectional study conducted by Ortega et al. [16] reported greater PA, measured by accelerometry, to be associated with self-reported longer sleep duration in children and adolescents. In another study investigating temporal associations between sleep and PA in children demonstrated that greater PA was negatively associated with objectively measured sleep health parameters, including shorter sleep durations and lower sleep efficiency [17]. Researchers of this study found that better sleep health parameters on a previous night were associated with lower PA the next day, which is aligned with the findings from the recent epidemiological studies [18, 19]. Such contradictory findings regarding the potential roles of PA on sleep health among adolescents have left knowledge gaps in the literature and require further investigation to elucidate their relationships.

There is also a growing body of literature demonstrating sedentary behavior (SED) as an emerging risk behavior negatively associated with various health outcomes including sleep health [20, 21]. Although the detailed physiological mechanisms as to how SED effects on sleep health are not yet fully understood, screen-based media use (i.e., screen time; SCT) is known to be one of the behavioral contexts of SED influencing sleep health in adolescents [14, 22]. Specifically, the displacement of good sleep hygiene (i.e., PA), increased physiological arousal by exposure to emotional contents, and delayed circadian rhythm due to the suppression of melatonin by exposure to bright light are possible mechanisms explaining the negative influence of SCT on sleep health [23]. SCT has been consistently reported to negatively associate with sleep health among school-aged children and adolescents [24, 25], with some studies reporting the bidirectionality of the associations across the years [26, 27]. This may suggest the possibility of day-to-day bidirectional associations between SCT and sleep health; however, there is a dearth of research that examined acute temporal associations between sleep health and SCT across school days in adolescents.

Currently, the evidence demonstrating temporal and bidirectional associations of PA and sleep health in adolescents is largely inconsistent [28]. There is also limited evidence for the bidirectional associations of SCT and sleep health. Given the increasing number of studies showing independence of SCT from SED [29, 30], it is necessary to examine SED and SCT with sleep health concurrently. Lastly, many previous studies have primarily focused on sleep-wake patterns such as sleep duration, efficiency, and onset latency. However, perceived sleep quality, which is thought to be orthogonal to sleep-wake patterns and considered one of the important indicators evaluating an individual's sleep health [19, 31], has received relatively little attention in the literature. Therefore, the primary purpose of this study was to examine the day-to-day bidirectional associations between PA, SED, including SCT, and sleep health parameters, including objectively measured sleep-wake patterns and perceived sleep quality in adolescents.

## Material and methods

### Participants

A convenience sampling method was used to recruit the participants from a public middle school located in the center of the medium-size city in Northwest Texas, USA. The school is a magnet school for Fine Arts, with a diverse student body of approximately seven hundred 6^th^ to 8^th^ graders attending from all over the city district. During the study period, the school started at 8:15 am until 4:00 pm. Under the school principal’s direction, a total of 315 students were accessible during general physical education classes. A brief verbal introduction to the study was provided in the class and a study information packet, including flyers, informed parental consent, and survey questionnaires that detailed demographics, medical, and health conditions were distributed. The eligibility criteria included: 1) without known physical or psychiatric disorders; 2) chronic medical conditions (e.g., asthma, diabetes, cancer); and 3) chronic sleep disorders. The Institutional Ethical Review Board at the Texas Tech University, TX, USA, approved the study protocols (approval#:505196) in accordance with the Declaration of Helsinki and its later amendments or comparable ethical standards.

### Procedures

Of the initially accessible students, 277 students met the eligibility criteria and agreed to participate in the study with the informed written parental consent as well as verbal assent. Those students were provided with a measurement packet comprising of the ActiGraph GT9X (ActiGraph, Inc., Pensacola, FL) accelerometer attached to a wristband, daily screen time diary, and sleep quality questionnaire. Students were asked 1) to wear the accelerometer on their non-dominant wrist for 24 hours whenever possible, except for water-involved activities, during the three consecutive school days; 2) to record the time if they remove the accelerometer; 3) to record daily screen times in the time diary each night before they go to bed; 4) to answer the sleep quality question in the packet each morning before they go to school; 5) to return the completed measurement packet to the school after monitoring periods. Parents were also asked to supervise and assist their child to comply with the measurement protocols each day. After data collection, 14 students who did not respond to survey questionnaires and/or provide valid accelerometer data (explained later) were further excluded. The final analytic sample included 263 students (boys: 135; mean age = 12.24 years), and descriptive characteristics of the sample are presented in Table 1.

**Table 1:** Descriptive characteristics of study participants.

	Total	Boys	Girls
n	263	135	128
Age (years)	12.24 (0.99)	12.10 (1.03)	12.39 (0.93)
Grade (n, %)			
6^th^	117 (44.49%)	69 (51.11%)	48 (37.50%)
7^th^	76 (28.90%)	40 (29.63%)	36 (28.13%)
8^th^	70 (26.62%)	26 (19.26%)	44 (34.38%)
Race/ethnicity (n, %)			
Non-Hispanic white	95 (36.12%)	45 (33.33%)	50 (39.06%)
Hispanic white	124 (47.15%)	68 (50.37%)	56 (43.75%)
Others	44 (16.73%)	22 (16.30%)	22 (17.19%)
Annual household income (n, %)			
<$35k	71 (28.51%)	32 (24.62%)	39 (32.77%)
$35k - <$75k	91 (36.55%)	49 (37.69%)	42 (35.29%)
≥$75k	87 (34.94%)	49 (37.69%)	38 (31.93%)
(missing)	14	5	9
Highest parental education			
≤ Highschool graduate	47 (18.36%)	16 (11.94%)	31 (25.41%)
Some college	82 (32.03%)	47 (35.07%)	35 (28.69%)
College graduate or more	127 (49.61%)	71 (52.99%)	56 (45.90%)
(missing)	7	1	6
Height (cm)	155.42 (10.14)	154.96 (12.02)	155.93 (7.61)
Weight (kg)	50.23 (13.71)	50.17 (15.98)	50.29 (10.77)
Body mass index (kg/m^2^) ^a^	20.61 (0.99)	20.65 (5.14)	20.56 (3.49)
Normal weight (n, %)	157 (64.88%)	78 (61.90%)	79 (68.10%)
Overweight/obese (n, %)	85 (35.12%)	48 (38.10%)	37 (31.90%)
(missing)	21	9	12

Values are mean (standard deviation) for continuous variables and n (%) for categorical variables.

^a^ the categorization was based on the CDC’s age- and sex-specific BMI growth chart (normal: >85^th^ percentile; and overweight/obese: ≥85^th^ percentile.

### Measures

#### Accelerometer-based physical activity and sleep-wake patterns

The accelerometer data collected from a wrist-worn ActiGraph GT9X accelerometer were downloaded using the ActiLife software (version 6.12.0; ActiGraph, Inc., Pensacola, FL). Accelerometer non-wear time was estimated using the Choi’s algorithm [32] in addition to the non-wear time log provided by the students. Choi's algorithm was set to detect minute-by-minute time intervals with at least 90-minutes of consecutive zero counts in the vertical axis, with an allowance of up to 2 minutes of interruptions if no activity counts are detected within 30-minutes of upstream and downstream from that interval. The estimated non-wear time was used to determine a valid accelerometer wear day that was defined as a day with at least 13 hours of wear time in waking hours. For the estimation of PA levels from the wrist-worn GT9X accelerometer, the activity counts at the vertical axis downloaded with normal filer at 5-second epoch length were used. The Chandler’s vertical cut points [33] were applied to estimate time spent in moderate and vigorous-intensity PA (MVPA) and SED during accelerometer wear time in a day. Time spent in MVPA and SED were extracted from the two segments of the day, before and after the school dismissal time at 4:00 pm for each day. The distinction of the day by the school dismissal time was based on the broad interest in the role of school and after-school hours in promoting PA and other health-related behaviors [24, 34]. The hours from wake-up time until school dismissal time was denoted as ‘during school hours’ and the hours from after school until bedtime was denoted as ‘after school hours’ through the paper.

The following sleep health parameters were estimated from the wrist-worn GT9X accelerometer using Sadeh's algorithm [35]: 1) total sleep time (i.e., actual sleep time); 2) sleep efficiency (%, total sleep time/total time in bed × 100); and 3) sleep fragment (%, number of awakening/total sleep time × 100). Sadeh’s algorithm was developed among children aged between 10 and 16 years old and demonstrated acceptable accuracy (sensitivity = 95% and specificity = 74.5%) to detect sleep-wake patterns against a gold standard measure of polysomnography [35].

#### Sleep quality

The perceived sleep quality on a previous night was measured using the four items modified from the previous studies [36, 37]: “*How would you rate your sleep quality at previous night overall*?”, “*Do you have difficulty falling asleep at previous night*?”, “*Do you wake up too early in this morning without being able to get back to sleep*?”, and “*Do you wake up feeling unrefreshed in this morning*?” using a 5-point Likert scale ranging from ‘very poor’ (most negative) to ‘very good’ (most positive) for the first item and from ‘extremely’ (most negative) to ‘not at all’ (most positive) for the remaining items. Pictorial assistance using a face emoticon was also provided for each response level (e.g., angry face for the most negative and big smiley face for the most positive). Students were asked to answer the sleep quality questions every morning before they go to the school and were supervised and assisted by their parents. Internal consistency was tested using a Cronbach’s alpha and ranged from 0.69 to 0.73 across monitoring days, which exceeded the lower limit of the acceptable level of internal consistency (≥0.60) [38]. An average sleep quality score was calculated across the four items and then log-transformed for normalization in data analyses.

#### Screen time diary

Daily time spent in SCT was measured using a self-report time-use diary, which has been successfully utilized in previous studies and highly recognized to demonstrate better measurement properties when comparing to questionnaire-based SCT measures [39, 40]. Students were provided with the daily time-use diary consisting of 15-minute slots for a 10-hour period after school hours (i.e., 4:00 pm– 2:00 am). Students were asked to mark and/or highlight the slots when they were engaged in screen-based media uses, with the assistance of their parents. In accordance with the screen-time questions from the U.S. Youth Risk Behavioral Surveillance [41], the students were specifically asked to record the time they spent in watching TV, playing computer or video games, and using a computer other than for school-related work after school hours, but without distinction of the types of screen-based media use. Time spent in SCT was calculated by adding all slots marked/highlighted for each day and then log-transformed for normalization in data analyses.

#### Other study covariates

Student’s age (years), grade, race/ethnicity (non-Hispanic white, Hispanic white, and others), annual household income (<$35k, $35k - <$75k, and ≥75k), and highest parental education (high school graduate, some college, and college graduate or more) were obtained from the parental questionnaire. Parent-reported student’s height (cm) and weight (kg) were used to calculate body mass index and the CDC’s age- and sex-specific growth chart [42] was applied to categorized students into the two groups (<85^th^ percentile: normal weight; and ≥85^th^ percentile: overweight/obese).

### Data analysis

Descriptive statistics of study variables were estimated using a linear mixed model accounting for repeated measures across the three consecutive school days. The estimated covariance parameters, including between- and within-subject variances, were used to calculate the intra-class correlation (ICC), which represents the day-to-day variability of study variables across the monitoring days by calculating the ratio of between-subject variability among the total variance of each study variable. Lower ICC indicates larger within-subject variability relative to between-subject variability. Additionally, bivariate correlations between study variables across the monitoring days were estimated using a linear mixed model based on the procedures described in Hamlett et al. [43] The confidence intervals of correlation coefficients were estimated from 100 bootstrapped samples drawn with replacement from the original observations.

Two mixed models with repeated measures were established to examine the day-to-day bidirectional associations between study variables: 1) the model predicting sleep health parameters on a night based on MVPA, SED, and SCT during that day; and 2) the model predicting MVPA, SED, and SCT on a day based on sleep health parameters the previous night, respectively. Given the 3 days of monitoring periods (4^th^-day return), there were three repeated observations of MVPA, SED, and SCT that predict sleep health parameters that night. In a reverse direction, there were two repeated observations of sleep health parameters available that predicts MVPA, SED, and SCT the next day. For each model, the within-subject variability by repeated measures was accounted by specifying the unstructured covariance structure of the residuals, which best fitted the data when compared to other covariance structures based on the model-data fit indices (e.g., lowest Akaike Information Criterion and Bayesian Information Criteria). The restricted maximum likelihood estimation was used for the parameter estimation from the final models adjusting for study covariates.

Additionally, autoregressive cross-lagged modeling was used to test temporal bidirectional associations of study variables across the school days. Unlike the mixed modeling approach that requires building the two separate models for each temporal direction of the association, the autoregressive cross-lagged model is capable to concurrently examine bidirectional associations of study variables in the framework of path analysis. In particular, the path model approach allows testing both direct and indirect associations between the variables across the entire course of monitoring days. For this analysis, we used total activity counts accumulated during waking hours for each day in replacement of MVPA and SED minutes, which allowed us to focus on daily activity levels irrespective of specific intensity levels while maintaining model simplicity. A priori hypothesized path model delineating bidirectional associations of total activity counts and SCT with sleep health across the three consecutive school days was established for each sleep health parameter. Model fit indices including the root mean square error of approximation (RMSEA), comparative fit index (CFI), and standardized root mean square residual (SRMR) were evaluate for the acceptability of the model (i.e., RMSEA<0.08; CFI≥.90; and SRMR <0.08) [44]. The maximum likelihood method was used for the parameter estimations including direct and indirect associations between study variables. The indirect associations were estimated by products of direct path coefficients and the associated standard error was estimated using the delta method [45].

The SAS v9.4 (SAS Institute Inc, Cary, NC) was used for the mixed model analyses and Mplus v7.2 (Muthen & Muthen, Los Angeles, CA) for the autoregressive cross-lagged modeling analysis. The statistical significance was set at ≤ .05.

## Results

Table 2 presents the descriptive statistics of study variables across the three monitoring days. On average, participants wore the accelerometer for 15.57 hours during waking time per each monitoring day. Average MVPA minutes observed during monitoring days ranged between 15.38 and 19.01 minutes during school hours and between 13.03 and 13.36 minutes after school hours, respectively. Self-reported SCT obtained from time-use diary ranged between 73.82 and 74.87 minutes and the estimated total sleep time ranged between 383.94 and 396.07 minutes across monitoring days. Relatively large within-subject variability (ICC < .50) was estimated from the most variables, with the largest within-subject variability observed from sleep fragment (ICC = .22). SCT showed the lowest within-subject variability with the largest ICC of .63, followed by SED_(during)_ (ICC = .58).

**Table 2:** Descriptive statistics of study variables across the three consecutive school days.

	Day 1	Day 2	Day 3	ICC^a^
Accelerometer wear time (hours)^b^	15.51 (15.33, 15.69)	15.62 (15.44, 15.80)	15.59 (15.41, 15.77)	.32
Activity counts (total)^c^	154.80 (149.74, 159.87)	165.67* (160.60, 170.75)	169.97* (164.89, 175.05)	.55
*During school hours*				
MVPA_(during)_ (mins)	15.38 (14.07, 16.68)	15.45 (14.14, 16.76)	19.01*^†^ (17.69, 20.32)	.48
SED_(during)_ (mins)	376.72 (369.78, 383.66)	356.46* (349.49, 363.43)	348.53* (341.56, 355.49)	.58
*After school hours*				
MVPA_(after)_ (mins)	13.07 (11.06, 15.08)	13.03 (11.02, 15.05)	13.36 (11.34, 15.38)	.31
SED_(after)_ (mins)	251.29 (242.53, 260.05)	256.57 (247.77, 265.37)	256.49 (247.68, 265.31)	.37
SCT (mins)^d^	98.76 (88.46, 109.06)	97.16 (86.86, 107.46)	97.17 (86.85, 107.50)	.63
*During a night*				
Total sleep time (min)	396.07 (387.44, 404.70)	396.11 (387.29, 404.92)	383.94 (374.75, 393.14)	.31
Sleep efficiency (%)	80.74 (79.81, 81.67)	81.02 (80.06, 81.97)	80.54 (79.55, 81.52)	.44
Sleep fragment (%)	24.69 (23.48, 25.90)	24.05 (22.81, 25.29)	24.89 (23.59, 26.18)	.22
Sleep quality^d^	3.98 (3.89, 4.08)	4.08 (3.98, 4.18)	4.10 (4.00, 4.20)	.60

ICC = intra-class correlation; MVPA = moderate- and vigorous-intensity physical activity; SED = sedentary behavior; SCT = screen time.

*Note*. values are least-square means (95% confidence intervals) estimated from a linear mixed model accounting multiple observation within each individual, unless otherwise specified.

* statistically different from day 1 at the *Bonferroni* adjusted alpha level of .017.

^†^ statistically different from day 2 at the *Bonferroni* adjusted alpha level of .017.

^a^ ICCs were calculated using covariance parameters including between- and within-subject variances estimated from a linear mixed model.

^b^ valid accelerometer wear time during waking hours.

^c^ total activity counts accumulated during wear hours were divided by 10,000 to reduce the unit size.

^d^ values are back transformed from log units.

The results of mixed model analyses examining temporal associations of MVPA, SED, and SCT during the day with sleep health parameters that night are presented in Table 3. Irrespective of the time of the day, MVPA minutes were not significantly associated with any of the sleep health parameters (*P*’s > .05). Greater SED minutes during school hours were associated with greater total sleep time (b = 2.51; *P* < .001) and sleep quality that night (b = 0.003; *P* = .016). Whereas, SED minutes accumulated after school hours were associated with lower total sleep time (b = -2.14; *P* < .001), greater sleep efficiency (b = 0.17; *P* = .005) and lower sleep fragment (b = -0.22; *P* = .004). Lastly, greater SCT was negatively associated with sleep quality (b = -0.010; *P* = .018).

**Table 3:** Temporal associations of physical activity, sedentary behavior and screen times during the day with sleep health parameters that night.

	Total sleep time (minutes)		Sleep efficiency (%)		Sleep fragment (%)		Sleep quality ^a^	
	b (95% CI)	*P*-value	b (95% CI)	*P*-value	b (95% CI)	*P*-value	b (95% CI)	*P*-value
*During school hours*								
MVPA_(during)_ ^b^	1.72 (-3.89, 7.34)	.547	-0.42 (-1.06, 0.20)	.188	0.48 (-0.36, 1.32)	.266	0.002 (-0.012, 0.016)	.746
SED_(during)_ ^b^	2.51 (1.52, 3.49)	< .001	0.08 (-0.03, 0.19)	.171	-0.05 (-0.20, 0.10)	.506	0.003 (0.001, 0.006)	.016
*After school hours*								
MVPA_(after)_ ^b^	-1.40 (-4.64, 1.84)	.397	0.23 (-0.13, 0.59)	.205	-0.36 (-0.85, 0.14)	.156	0.005 (-0.003, 0.013)	.215
SED_(after)_ ^b^	-2.14 (-3.15, -1.12)	< .001	0.17 (0.06, 0.29)	.005	-0.22 (-0.38, -0.07)	.004	0.002 (-0.000, 0.005)	.150
SCT ^a^	-1.16 (-4.31, 2.00)	.472	-0.19 (-0.55, 0.17)	.297	0.36 (-0.10, 0.83)	.123	-0.010 (-0.019, -0.002)	.018

b = unstandardized regression coefficient; CI = confidence interval; MVPA = moderate- and vigorous-intensity physical activity; SED = sedentary behavior; SCT = screen time.

*Note*. the estimates are from a linear mixed model established for each sleep health parameter adjusting for study covariates including gender, grade, race, and body mass index group.

^a^ log-transformed.

^b^ the estimates were multiplied by 10 to indicate the changes in sleep health parameters by 10-min increments in MVPA and SED minutes, respectively.

Table 4 presents the results of mixed model analyses examining temporal associations of sleep health parameters on a previous night with MVPA, SED, and SCT the next day. Greater total sleep time on the previous night was associated with lower SED minutes the next day (b = -10.66; *P* < .001 for during school hours; and b = -5.88; *P* = .001 for after school hours). Sleep efficiency was positively associated with SED minutes accumulated during school hours the next day (b = 1.33; *P* = .006). Lastly, greater sleep quality on the previous night was associated with greater MVPA minutes during the school hours (b = 6.45; *P* = .028) and lower SED minutes after school hours the next day (b = -39.85; *P* = .029).

**Table 4:** Temporal associations of sleep health parameters on the previous night with physical activity, sedentary behavior and screen time the next day.

	During school hours				After school hours					
	MVPA_(during)_		SED_(during)_		MVPA_(after)_		SED_(after)_		SCT ^a^	
	b (95% CI)	*P*	b (95% CI)	*P*	b (95% CI)	*P*	b (95% CI)	*P*	b (95% CI)	*P*
Total sleep time ^b^	0.36 (-0.20, 0.92)	.211	-10.66 (-13.33, -7.98)	< .001	0.16 (-0.70, 1.02)	.711	-5.88 (-9.44, -2.33)	.001	0.0002 (-0.002, 0.002)	.806
Sleep efficiency	-0.04 (-0.24, 0.16)	.693	1.33 (0.39, 2.28)	.006	-0.06 (-0.36, 0.25)	.713	0.78 (-0.49, 2.05)	.225	0.005 (-0.02, 0.03)	.706
Sleep fragment	2.43 (-1.75, 6.61)	.254	-3.18 (-22.48, 16.12)	.746	3.82 (-2.73, 10.36)	.252	-25.63 (-52.89, 1.63)	.065	-0.004 (-0.02, 0.01)	.639
Sleep quality ^a^	6.45 (0.70, 12.21)	.028	-23.62 (-51.99, 4.74)	.102	8.43 (-0.24, 17.09)	.057	-39.85 (-75.6, -4.10)	.029	-0.49 (-1.29, 0.32)	.237

MVPA = moderate- and vigorous-intensity physical activity; SED = sedentary behavior; SCT = screen time; b = unstandardized regression coefficient; *P* = *P*-value

*Note*. the estimates are obtained from a linear mixed model adjusting for study covariates including gender, grade, race, and body mass index group.

^a^ log-transformed.

^b^ the estimates are multiplied by 30 to indicate the changes in sleep health parameters by 30-min increments in total sleep time with an exception for the model predicting SCT that was log-transformed.

**Fig 1:**
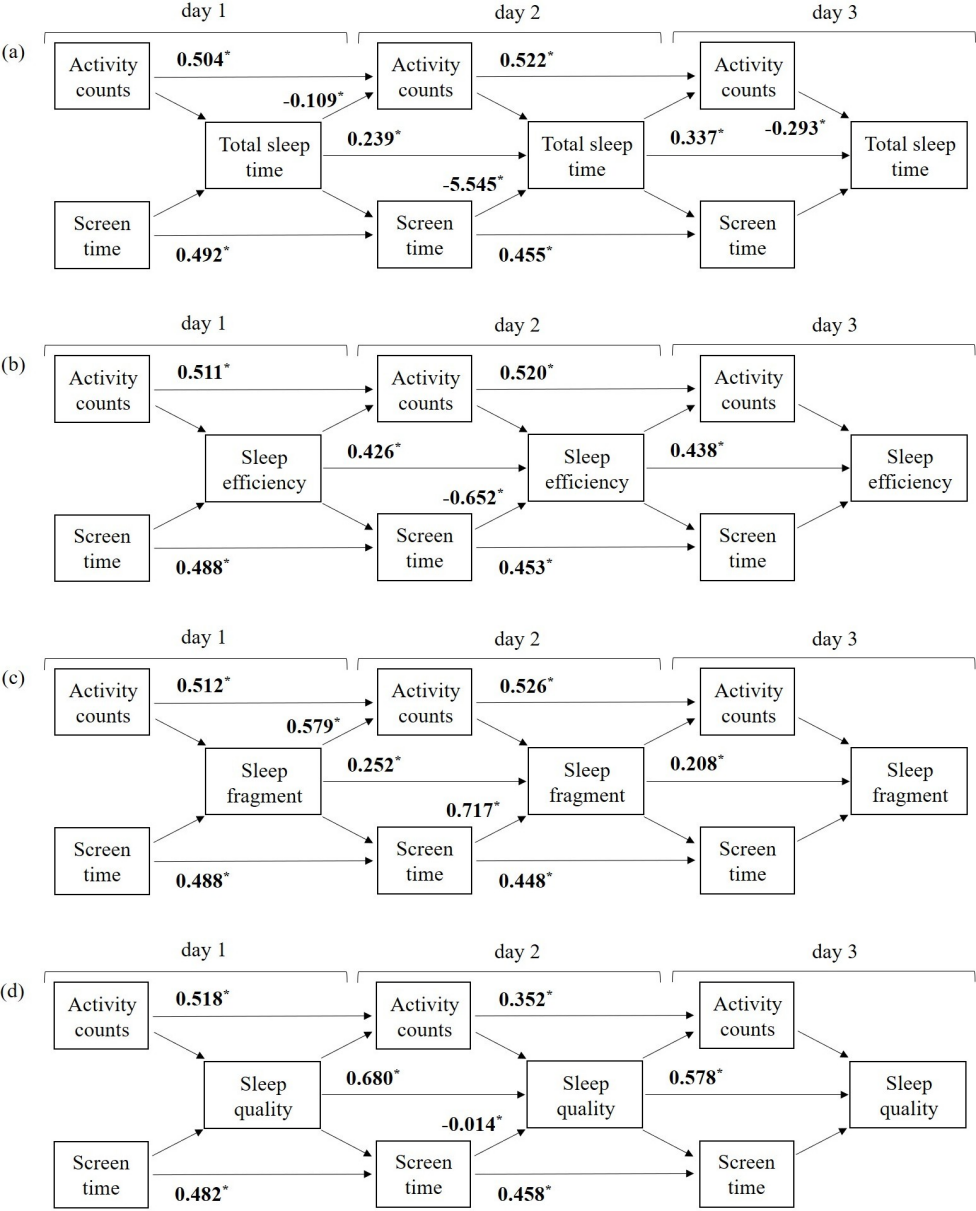
An autoregressive cross-lagged path model examining temporal and bidirectional associations between daily activity counts, SCT, and following sleep health parameters across the three school days: (a) total sleep time; (b) sleep efficiency; (c) sleep fragment; and (d) sleep quality. Screen time and sleep quality were log-transformed, and activity counts were multiplied by 10,000 to reduce the unit size. Only significant path coefficients are boldly reported in the form of the unstandardized coefficient. Complete results are reported in S2 Table through 5 for each sleep health outcome.

Fig 1 depicts the path diagrams of the autoregressive cross-lagged model examined for each sleep health parameter (note. the complete results are presented in the S2–S5 Tables). The results highlighted significant day-to-day autoregressive effects for all study variables, where activity counts, SCT, and sleep health parameters on the day were consistently associated with greater levels of respective attributes the next day. Pertaining to bidirectional associations between activity counts and sleep health parameters, the results showed no significant associations of activity counts during the day with sleep health parameters that night across the entire monitoring days, with an exception of day 3 where greater activity counts during the day were associated with lower total sleep time that night (b = -0.293; *P* = .012). In the opposite direction, total sleep time in the day 1 night was negatively associated with activity counts in day 2 (b = -0.109; *P* < .001) and, during the same time, greater sleep fragment was positively associated with activity counts (b = 0.579; *P* = .019). There were no significant bidirectional associations between activity counts and sleep efficient and sleep quality across the entire monitoring days (*P*’s >.05).

There were no temporal associations of sleep health parameters on the night with SCT the next day across the monitoring days (*P*’s > .05). In the opposite direction, there were no temporal associations of SCT with sleep health parameters observed during the time in day 1 and day 3; yet, consistent associations were observed during day 2. The results showed that greater SCT on day 2 were significantly and unfavorably associated with all of the sleep health parameters that night (b = -5.545; *P* = .014 for total sleep time, b = -0.652; *P* = .008 for sleep efficiency, b = 0.717; *P* = .034 for sleep fragment, and b = -0.014; *P* = .021 for sleep quality).

The results of follow-up analyses testing indirect associations between the study variables are presented in Table 5. There were significant indirect associations of SCT with all of sleep health parameters across the days throughout the various paths. For instance, SCT on day 1 was indirectly associated with total sleep time in day 2 night via SCT in day 2 (Screen time_(day 1)_ → Screen time_(day 2_) → Total sleep time_(day 2)_: b = -2.727, *P* = .018). However, there were no indirect, bidirectional associations of sleep health parameters with SCT the next day. Meanwhile, there were significant bidirectional indirect associations between activity counts and total sleep time, where the associations negatively influenced each other across the days. For instance, greater total sleep time in day 1 night negatively associated with activity counts in day 3 vis activity counts in day 2 (Total sleep time_(day 1)_ → Activity counts_(day 2)_ → Activity counts_(day 3)_; b = -0.057; *P* = .001).

**Table 5:** Significant indirect associations estimated from autoregressive cross-lagged path models.

Indirect path	b (95% CI)	*P*-value
*Screen time → Total sleep time*^a^		
Screen time_(day 1)_ → Screen time_(day 2)_ → Total sleep time _(day 2)_	-2.727 (-4.981, -0.473)	.018
Screen time_(day 1)_ → Screen time_(day 2)_ → Total sleep time _(day 2)_ → Total sleep time_(day 3)_	-0.919 (-1.764, -0.074)	.033
Screen time_(day 2)_ → Total sleep time_(day 2)_ → Total sleep time_(day 3)_	-1.868 (-3.540, -0.196)	.028
*Activity counts → Total sleep time*^a^		
Activity counts_(day 1)_ → Activity counts_(day 2)_ → Activity counts_(day 3)_ → Total sleep time_(day 3)_	-0.077 (-0.142, -0.012)	.018
Activity counts_(day 2)_ → Activity counts_(day 3)_ → Total sleep time_(day 3)_	-0.153 (-0.276, -0.030)	.015
*Total sleep time → Activity counts*^a^		
Total sleep time_(day 1)_ → Activity counts_(day 2)_ → Activity counts_(day 3)_	-0.057 (-0.090, -0.024)	.001
*Screen time → Sleep efficiency*^b^		
Screen time_(day 1)_ → Screen time_(day 2)_ → Sleep efficiency_(day 3)_	-0.318 (-0.561, -0.075)	.010
Screen time_(day 1)_ → Screen time_(day 2)_ → Sleep efficiency_(day 2)_ → Sleep efficiency_(day 3)_	-0.139 (-0.253, -0.025)	.017
Screen time_(day 2)_ → Sleep efficiency_(day 2)_ → Sleep efficiency_(day 3)_	-0.285 (-0.510, -0.060)	.013
*Screen time → Sleep fragment*^c^		
Screen time_(day 1)_ → Screen time_(day 2)_ → Sleep fragment_(day 2)_	0.350 (0.019, 0.681)	.039
*Screen time → Sleep quality*^d^		
Screen time_(day 1)_ → Screen time_(day 2)_ → Sleep quality_(day 2)_	-0.007 (-0.013, -0.001)	.025
Screen time_(day 1)_ → Screen time_(day 2)_ → Sleep quality_(day 2)_ → Sleep quality_(day 3)_	-0.004 (-0.008, -0.0001)	.028
Screen time_(day 2)_ → Sleep quality_(day 2)_ → Sleep quality_(day 3)_	-0.008 (-0.016, -0.0002)	.024

b = unstandardized path coefficient; CI = confidence interval.

^a^ estimated from the model (a) presented in the Fig 1.

^b^ estimated from the model (b) presented in the Fig 1.

^c^ estimated from the model (c) presented in the Fig 1.

^d^ estimated from the model (d) presented in the Fig 1.

## Discussion

This study examined the day-to-day bidirectional associations between MVPA, SED, SCT, and sleep health in adolescents during school days. Our findings highlighted that MVPA minutes in a day, regardless of the time segment where MVPA minutes were accumulated, was not a significant predictor of sleep health on that night. Similarly, MVPA minutes in a day were not influenced by objectively measured sleep-wake patterns the previous night, but perceived sleep quality was the significant predictor of greater MVPA the next day. The bidirectional associations were more apparent between SED and sleep health, but the significance and direction of the associations varied by the time segment of a day where SED was accumulated as well as types of sleep health parameters. Additionally, greater SCT during the day was associated with lower sleep quality that night, and greater sleep quality was associated with lower SED after school hours the next day. Lastly, there were significant indirect associations of SCT with sleep health parameters across the days indicating multi-day lagged effects of SCT on sleep health.

It has been frequently reported that PA leads to better sleep in adolescents. For instance, a recent observational study of 417 adolescents demonstrated that greater MVPA in a day is associated with longer sleep duration and greater sleep efficiency [18]. Their results are also aligned with a recent experimental study showing that a 12-week supervised exercise program consisting of aerobic and resistance training weekly improved sleep duration and sleep efficiency in adolescents [46]. The present study, however, found the null association between MVPA and sleep health parameters including both sleep-wake patterns and perceived sleep quality, which is concordant with the growing evidence from epidemiological studies [47–50]. Path model analyses based on activity counts further demonstrated no temporal associations with sleep health parameters across the monitoring days. Such contradictory findings related to the temporal effects of PA on sleep health have also been reported in different age groups; yet, the underlying reasons are not fully understood. Kline [51] reported that the positive effects of exercise on sleep are more consistently reported among studies, including individuals with more severe chronic sleep problems than those with normal sleepers. The sample of the present study was restricted to adolescents without chronic sleep disorders to avoid possible confounding effects of clinical sleep treatment (e.g., medications or therapies) in the observational results. However, such exclusion criteria might explain the null associations of PA with sleep health observed from the present study, possibly due to the ceiling effect [52, 53].

Meanwhile, there have been several studies that examined the possible moderating factors influencing the effects of PA on sleep health, which include, but are not limited to, time of the day, intensity, and duration of PA. A recent meta-analysis of 23 experimental studies reported that engaging in high-intensity exercise very close to bedtime negatively impacts sleep health, including delayed sleep-onset and reduced total sleep time [54]. Other experimental studies found that, however, engaging in late-night exercise at moderate to vigorous intensities did not influence sleep health among physically fit adults [55, 56], indicating the possible mediating role of individual’s fitness level as greater fitness level is associated with shorter recovery of exercise-induced sympathetic nervous system arousal [57]. The present study did not examine PA levels during specific hours before bedtime in a day due to the difficulty in processing accelerometer-determined bedtime data that vary by individual. Rather, we examined PA levels accumulated during the two segments of a day, during and after school hours, resulting in null associations. Given that most evidence showing possible moderating effects of intensity of PA and time of the day on the relationship of PA with sleep health have been reported in different age groups, further studies are needed among the adolescent population.

Additionally, our findings based on the mixed model analyses showed no significant associations of objectively measured sleep-wake patterns on the night with MVPA levels the next day. These results are in line with some but not all findings previously reported from other observational studies that utilized similar designs and methods in the pediatric population [17, 48, 58]. As previously noted, there is a large variation in the reported associations between sleep health and PA levels in the current body of literature. For instance, Lin et al. [58] found greater sleep duration be associated with greater MVPA minutes on the following day among the international sample of 5779 children aged 9–11 years from the 12 countries. Notably, the authors also found that, in the stratified analyses by geographical areas, favorable associations disappeared among subsamples from some countries; yet, the underlying reasons were not clear. Vincent et al.[48] reported that such discrepancies might be attributed to differences in sleep duration and PA levels between study samples. However, their results showing null associations of total sleep time with PA levels were supported by the present study, despite that our sample had significantly shorter sleep duration (<400 minutes/day) and lower MVPA levels (<30 minutes/day) than their sample (sleep time: ≈475 minutes/day; MVPA: 121 minutes/day). Thus, it remains unclear whether differences in sleep time and PA levels affect those relationships, and further research examining possible moderating factors explaining differences in bidirectional associations between sleep health and activity levels in adolescents is necessary.

Our findings were consistent with previous studies [47, 58] that demonstrated significant bidirectional associations between sleep health and SED. Notably, each study varied by the types of sleep health parameters and time of the day from which SED was accumulated. We found that greater SED accumulated during school hours was associated with longer sleep duration as well as sleep quality that night. Whereas SED accumulated after school hours was associated with shorter sleep durations but with better sleep parameters (e.g., greater sleep efficiency and lower sleep fragment). In contrast, longer sleep duration on the previous night was inversely associated with SED the following day, but better sleep efficiency was associated with greater SED the next day. Such contradictory results are not clearly understood in the present study, but there are few plausible explanations for some findings. The inverse association of SED accumulated after school hours with sleep durations may be due to the deficit of total sleep duration possibly replaced into SED. Another explanation may be related to circadian typology (i.e., morningness and eveningness), which is known to influence the relationship between sleep and daytime behaviors in adolescents [59]. Likewise, the inverse association of sleep durations with SED the next day may also be due to the decreases in total waking time as a result of increased sleep durations; yet the magnitude of associations (e.g., 2.11 minutes changes in sleep durations in response to 10-minute changes in SED) may not be meaningful [58].

The present study expands the current body of literature by examining the role of SCT in sleep health during school days. The findings based on mixed model analyses demonstrated that greater SCT in the day was associated with lower perceived sleep quality that night. The results of follow-up analyses based on the path model approach showed that, however, greater SCT was negatively associated with not only the perceived sleep quality but also objectively measured sleep-wake patterns; yet, the significances of the associations were only apparent in day 2. It is difficult to directly compare the results obtained from the different analytic models focusing different level of interests (e.g., average daily associations for mixed model vs., single day association for path model), but our results are partially aligned with the current understanding of negative associations of SCT with sleep health parameters [23, 59]. In particular, we further observed significant indirect associations of SCT with both objectively measured sleep-wake patterns and perceived sleep quality across the multiple days, showing the evidence of multi-day lagged-effects of SCT on sleep health the later nights. Collectively, our study adds to the existing literature highlighting the importance of reducing SCT for better sleep health in adolescents, which, at large, may support the hypothesis of the mediating role of sleep health on the detrimental effects of SCT on various health outcomes [60, 61].

Meanwhile, it has been previously reported that the significance and magnitude of associations between SCT and sleep health parameters may vary depending on the types and measurement methods of SCT [24, 62]. In the present study, SCT was measured using the time-diary method, which is known to improve measurement properties of SCT compared to questionnaire-based methods [39], but without consideration of types of SCT. Thus, our results should be interpreted in terms of overall SCT, including watching TV, playing computer or video games, and using a computer other than for school-related work, which might attribute null associations observed in this study. Additionally, time of the day in which SCT was accumulated such as playing video games close to the bedtime hours or use of portable devices in the bed could be other possible moderators influencing temporal associations of SCT with sleep health parameters [62, 63]. Further research is suggested to examine differential effects on bidirectional associations between SCT and sleep health by potential moderators.

Perceived sleep quality is one of the important sleep health parameters associated with various daytime functional and behavioral outcomes independent of sleep-wake patterns [31, 64]. Based on path model analyses, the present study found that there were no significant associations of sleep quality with overall activity levels the next day across the entire monitoring periods. However, we also observed that, on average, greater sleep quality in the night was significantly associated with greater MVPA minutes during school hours as well as lower SED minutes after school hours. This implies that sleep quality on the previous night may dissimilarly affect behavioral outcomes the next day by the different time segments of the day.

The present study has several strengths, such as the utilization of objectively methods quantifying sleep-wake patterns as well as PA levels, expansion of measurement scope including SCT and perceived sleep quality, and nature of research questions examining temporal bidirectional associations across school days. However, several limitations should be accounted for when interpreting the present findings. First, the length of monitoring time was relatively short (4 days and three nights in a single week), which affects the generalizability of the present results. Cross-validations of the observed associations across multiple weeks using multilevel analytic approach (i.e., multiple monitoring days nested within multiple weeks), are suggested for future research. Second, the present study did not include all possible moderating and/or mediating factors affecting the observed associations. Specifically, in addition to factors previously discussed, the lack of control for dietary behaviors such as consumption of sugar-sweetened beverages close to bedtime might confound the observed results from this study. Third, the objectively measured sleep-wake patterns and PA levels are not free from measurement errors due to the indirect nature of estimating human behaviors and energy expenditures based on ambulatory movements, and thus, caution is needed when interpreting the results. Lastly, the study sample was limited to students attending the same school in the same geographical location, which may attribute to relatively low between-subject variability of study variables, possibly due to the similarity of daily routines during school days. Further, the sample size may not be adequately powered to address bidirectional associations examined in our primary analyses. We suggest that future research be conducted based on a larger sample with varying daily routines.

## Conclusion

In conclusion, the present study demonstrated a lack of temporal bidirectional associations of objectively measured MVPA and sleep-wake patterns among adolescents. Lower SCT during the day was associated with greater perceived sleep quality that night, which may positively affect daily behavioral outcomes during school days including greater MVPA levels during school hours and lower SED levels after school hours. SCT was also indirectly associated with unfavorable outcomes of objectively measured sleep-wake patterns the later nights, highlighting the importance of limiting SCT to improve sleep health in this vulnerable population. A future study aiming to develop and implement an intervention should consider including the reduction of SCT and improve perceived sleep quality as one of the key sleep hygiene strategies.

## Supporting information

S1 Table Correlations Between MVPA, Sedentary Behavior, Screen Time, and Sleep Health Parameters. (DOCX)

S2 Table Autoregressive Cross-Lagged Path Model Analysis with Total Sleep Time. (DOCX)

S3 Table Autoregressive Cross-Lagged Path Model Analysis with Sleep Efficiency. (DOCX)

S4 Table Autoregressive Cross-Lagged Path Model Analysis with Sleep Fragment. (DOCX)

S5 Table Autoregressive Cross-Lagged Path Model Analysis with Sleep Quality. (DOCX)
