# Supplementary material for: Examining the day-to-day bidirectional associations between physical activity, sedentary behavior, screen time, and sleep health during school days in adolescents
Source: PLoS One. 2020 Sep 3;15(9):e0238721. doi: 10.1371/journal.pone.0238721 (PMC7470331; doi:10.1371/journal.pone.0238721)
Supplement: S1 Table — (DOCX) [file pone.0238721.s001.docx]

**Supplement Table 1.**

**Correlations Between MVPA, Sedentary Behavior, Screen Time, and Sleep Health Parameters**

|  | During school hours | | After school hours | | | During a night | | | |
| --- | --- | --- | --- | --- | --- | --- | --- | --- | --- |
|  | MVPA_(during)_ | SED_(during)_ | MVPA_(after)_ | SED_(after)_ | SCT | Total  sleep time | Sleep efficiency | Sleep  fragment | Sleep  Quality |
| *During school hours* | |  |  |  |  |  |  |  |  |
| SED_(during)_ | -.38  (-.59, -.17) | - |  |  |  |  |  |  |  |
| *After school hours* | |  |  |  |  |  |  |  |  |
| MVPA_(after)_ | .18  (-.09, .45) | -.10  (-.36, .17) | - |  |  |  |  |  |  |
| SED_(after)_ | -.21  (-.47, .05) | .19  (-.08, .46) | -.27  (-.53, -.01) | - |  |  |  |  |  |
| SCT | -.10  (-.39, .19) | .10  (-.24, .45) | -.09  (-.38, .20) | .18  (-.12, .47) | - |  |  |  |  |
| *During a night* |  |  |  |  |  |  |  |  |  |
| Total sleep time | .02  (-.27, .30) | -.03  (-.33, .28) | .00  (-.26, .27) | -.34  (-.63, -.05) | -.08  (-.41, .25) | - |  |  |  |
| Sleep efficiency | -.09  (-.38, .19) | .07  (-.19, .34) | -.03  (-.27, .22) | .14  (-.19, .48) | .01  (-.34, .32) | .51  (.22, .80) | - |  |  |
| Sleep fragment | .05  (-.27, .37) | -.04  (-.30, .22) | .01  (-.26, .27) | -.15  (-.43, .14) | .01  (-.29, .31) | -.33  (-.63, -.04) | -.69  (-.85, -.52) | - |  |
| Sleep quality | .00  (-.37, .38) | .02  (-.28, .32) | .08  (-.23, .38) | -.05  (-.39, .29) | -.18  (-.52, .18) | .10  (-.22, .41) | .00  (-.32, .33) | -.08  (-.42, .26) |  |
| Activity counts | .57  (.38, .76) | -.42  (-.66, -.17) | .58  (.39, .77) | -.24  (-.61, .13) | -17  (-.55, .21) | -.14  (-.46, .20) | -.11  (-.40, .18) | .05  (-.28, .38) | .02  (-.39, .42) |

MVPA = moderate- and vigorous-intensity physical activity; SED = sedentary behavior; SCT = screen time.

*Note.* Values are correlation coefficients (95% confidence intervals) between each variable in a day estimated from a linear mixed model accounting for multiple observations within each individual. The confidence intervals were estimated from 100 bootstrapped samples drawn with replacement from the original observations.
