# Supplementary material for: Examining the day-to-day bidirectional associations between physical activity, sedentary behavior, screen time, and sleep health during school days in adolescents
Source: PLoS One. 2020 Sep 3;15(9):e0238721. doi: 10.1371/journal.pone.0238721 (PMC7470331; doi:10.1371/journal.pone.0238721)
Supplement: S2 Table — (DOCX) [file pone.0238721.s002.docx]

**Supplement Table 2.**

| Temporality of association | b | 95% CI | | *P*-value |
| --- | --- | --- | --- | --- |
|  |  | Lower | Upper |  |
| *(Day 1) Cross-lagged associations* |  |  |  |  |
| Activity counts_(day 1)_ → Total sleep time_(day 1)_ | -0.127 | -0.366 | 0.112 | .296 |
| Screen time_(day 1)_ → Total sleep time_(day 1)_ | 1.488 | -4.280 | 7.256 | .613 |
| *(Day 1 → Day 2) Cross-lagged associations* |  |  |  |  |
| Total sleep time_(day 1)_ → Activity counts_(day 2)_ | **-0.109** | **-0.703** | **0.485** | **<.001** |
| Total sleep time_(day 1)_ → Screen time_(day 2)_ | 0.0002 | -0.0002 | 0.0002 | .699 |
| *(Day 1 → Day 2) Lagged association* |  |  |  |  |
| Total sleep time_(day 1)_ → Total sleep time_(day 2)_ | **0.239** | **0.141** | **0.337** | **<.001** |
| Activity counts_(day 1)_ → Activity counts_(day 2)_ | **0.504** | **0.396** | **0.612** | **<.001** |
| Screen time_(day 1)_ → Screen time_(day 2)_ | **0.492** | **0.386** | **0.598** | **<.001** |
| *(Day 2) Cross-lagged associations* |  |  |  |  |
| Activity counts_(day 2)_ → Total sleep time_(day 2)_ | -0.080 | -0.264 | 0.104 | .397 |
| Screen time_(day 2)_ → Total sleep time_(day 2)_ | **-5.545** | **-9.975** | **-1.115** | **.014** |
| *(Day 2 → Day 3) Cross-lagged associations* |  |  |  |  |
| Total sleep time_(day 2)_ → Activity counts_(day 3)_ | -0.019 | -0.097 | 0.059 | .641 |
| Total sleep time_(day 2)_ → Screen time_(day 3)_ | 0.001 | -0.001 | 0.003 | .629 |
| *(Day 2 → Day 3) Lagged association* |  |  |  |  |
| Total sleep time_(day 2)_ → Total sleep time_(day 3)_ | **0.337** | **0.200** | **0.474** | **<.001** |
| Activity counts_(day 2)_ → Activity counts_(day 3)_ | **0.522** | **0.418** | **0.626** | **<.001** |
| Screen time_(day 2)_ → Screen time_(day 3)_ | **0.455** | **0.353** | **0.557** | **<.001** |
| *(Day 3) Cross-lagged associations* |  |  |  |  |
| Activity counts_(day 3)_ → Total sleep time_(day 3)_ | **-0.293** | **-0.520** | **-0.066** | **.012** |
| Screen time_(day 3)_ → Total sleep time_(day 3)_ | -4.483 | -9.771 | 0.805 | .097 |
| *Covariance^a^* |  |  |  |  |
| Activity counts_(day 1)_ ↔ Screen time_(day 1)_ | -5.942 | -14.176 | 2.292 | .157 |
| Activity counts_(day 2)_ ↔ Screen time_(day 2)_ | **-9.960** | **-16.869** | **-3.051** | **.005** |
| Activity counts_(day 3)_ ↔ Screen time_(day 3)_ | -6.039 | -12.405 | 0.327 | .063 |
| Screen time_(day 1)_ ↔ Screen time_(day 3)_ | **1.019** | **0.678** | **1.360** | **<.001** |
| Model data fit indices:  *x*^2^_(16)_ = 57.11(*P* <.001); RMSEA = .099 (.072, .127); CFI = .918; TLI = .821; SRMR = .053 | | | | |

**Autoregressive Cross-Lagged Path Model Analysis with Total Sleep Time.**

b = unstandardized path coefficient; CI = confidence interval; RMSEA = root mean square error of approximation; CFI = comparative fit index; TLI = Tucker Lewis index

*Note.* Bold indicates statistically significant effects.

^a^ covariance between ‘activity counts_(day 1)_’ and ‘activity counts_(day 3)_’ was fixed to zero due to non-convergence of the model.
