# Supplementary material for: Examining the day-to-day bidirectional associations between physical activity, sedentary behavior, screen time, and sleep health during school days in adolescents
Source: PLoS One. 2020 Sep 3;15(9):e0238721. doi: 10.1371/journal.pone.0238721 (PMC7470331; doi:10.1371/journal.pone.0238721)
Supplement: S3 Table — (DOCX) [file pone.0238721.s003.docx]

**Supplement Table 3.**

**Autoregressive Cross-Lagged Path Model Analysis with Sleep Efficiency.**

| Temporality of association | b | 95% CI | | *P*-value |
| --- | --- | --- | --- | --- |
|  |  | Lower | Upper |  |
| *(Day 1) Cross-lagged associations* |  |  |  |  |
| Activity counts_(day 1)_ → Sleep efficiency_(day 1)_ | -0.019 | -0.043 | 0.005 | .128 |
| Screen time_(day 1)_ → Sleep efficiency_(day 1)_ | 0.237 | -0.349 | 0.823 | .429 |
| *(Day 1 → Day 2) Cross-lagged associations* |  |  |  |  |
| Sleep efficiency_(day 1)_ → Activity counts_(day 2)_ | -0.433 | -1.025 | 0.159 | .152 |
| Sleep efficiency_(day 1)_ → Screen time_(day 2)_ | 0.009 | -0.015 | 0.033 | .455 |
| *(Day 1 → Day 2) Lagged association* |  |  |  |  |
| Sleep efficiency_(day 1)_ → Sleep efficiency_(day 2)_ | **0.426** | **0.326** | **0.526** | **<.001** |
| Activity counts_(day 1)_ → Activity counts_(day 2)_ | **0.511** | **0.399** | **0.623** | **<.001** |
| Screen time_(day 1)_ → Screen time_(day 2)_ | **0.488** | **0.382** | **0.594** | **<.001** |
| *(Day 2) Cross-lagged associations* |  |  |  |  |
| Activity counts_(day 2)_ → Sleep efficiency_(day 2)_ | -0.015 | -0.035 | 0.005 | .133 |
| Screen time_(day 2)_ → Sleep efficiency_(day 2)_ | **-0.652** | **-1.132** | **-0.172** | **.008** |
| *(Day 2 → Day 3) Cross-lagged associations* |  |  |  |  |
| Sleep efficiency_(day 2)_ → Activity counts_(day 3)_ | -0.252 | -0.920 | 0.416 | .460 |
| Sleep efficiency_(day 2)_ → Screen time_(day 3)_ | 0.006 | -0.016 | 0.028 | .615 |
| *(Day 2 → Day 3) Lagged association* |  |  |  |  |
| Sleep efficiency_(day 2)_ → Sleep efficiency_(day 3)_ | **0.438** | **0.313** | **0.563** | **<.001** |
| Activity counts_(day 2)_ → Activity counts_(day 3)_ | **0.520** | **0.416** | **0.624** | **<.001** |
| Screen time_(day 2)_ → Screen time_(day 3)_ | **0.453** | **0.351** | **0.555** | **<.001** |
| *(Day 3) Cross-lagged associations* |  |  |  |  |
| Activity counts_(day 3)_ → Sleep efficiency_(day 3)_ | -0.012 | -0.036 | 0.012 | .344 |
| Screen time_(day 3)_ → Sleep efficiency_(day 3)_ | 0.032 | -0.531 | 0.595 | .911 |
| *Covariance^a^* |  |  |  |  |
| Activity counts_(day 1)_ ↔ Screen time_(day 1)_ | -6.120 | -6.236 | -6.004 | .146 |
| Activity counts_(day 2)_ ↔ Screen time_(day 2)_ | **-9.609** | **-9.731** | **-9.487** | **.008** |
| Activity counts_(day 3)_ ↔ Screen time_(day 3)_ | -5.993 | -6.118 | -5.868 | .065 |
| Screen time_(day 1)_ ↔ Screen time_(day 3)_ | **1.026** | **0.914** | **1.138** | **<.001** |
| Model data fit indices:  *x*^2^_(16)_ = 64.93(*P* <.001); RMSEA = .108 (.081, .136); CFI = .911; TLI = .806; SRMR = .054 | | | | |

b = unstandardized path coefficient; CI = confidence interval; RMSEA = root mean square error of approximation; CFI = comparative fit index; TLI = Tucker Lewis index

*Note.* Bold indicates statistically significant effects.

^a^ covariance between ‘activity counts_(day 1)_’ and ‘activity counts_(day 3)_’ was fixed to zero due to non-convergence of the model.
