# Supplementary material for: Examining the day-to-day bidirectional associations between physical activity, sedentary behavior, screen time, and sleep health during school days in adolescents
Source: PLoS One. 2020 Sep 3;15(9):e0238721. doi: 10.1371/journal.pone.0238721 (PMC7470331; doi:10.1371/journal.pone.0238721)
Supplement: S4 Table — (DOCX) [file pone.0238721.s004.docx]

**Supplement Table 4.**

**Autoregressive Cross-Lagged Path Model Analysis with Sleep Fragment.**

| Temporality of association | b | 95% CI | | *P*-value |
| --- | --- | --- | --- | --- |
|  |  | Lower | Upper |  |
| *(Day 1) Cross-lagged associations* |  |  |  |  |
| Activity counts_(day 1)_ → Sleep fragment_(day 1)_ | 0.013 | -0.016 | 0.042 | .403 |
| Screen time_(day 1)_ → Sleep fragment_(day 1)_ | -0.242 | -0.957 | 0.473 | .507 |
| *(Day 1 → Day 2) Cross-lagged associations* |  |  |  |  |
| Sleep fragment_(day 1)_ → Activity counts_(day 2)_ | **0.579** | **0.093** | **1.065** | **.019** |
| Sleep fragment_(day 1)_ → Screen time_(day 2)_ | -0.007 | -0.027 | 0.013 | .473 |
| *(Day 1 → Day 2) Lagged association* |  |  |  |  |
| Sleep fragment_(day 1)_ → Sleep fragment_(day 2)_ | **0.252** | **0.136** | **0.368** | **<.001** |
| Activity counts_(day 1)_ → Activity counts_(day 2)_ | **0.512** | **0.402** | **0.622** | **<.001** |
| Screen time_(day 1)_ → Screen time_(day 2)_ | **0.488** | **0.382** | **0.594** | **<.001** |
| *(Day 2) Cross-lagged associations* |  |  |  |  |
| Activity counts_(day 2)_ → Sleep fragment_(day 2)_ | 0.016 | -0.011 | 0.043 | .241 |
| Screen time_(day 2)_ → Sleep fragment_(day 2)_ | **0.717** | **0.055** | **1.379** | **.034** |
| *(Day 2 → Day 3) Cross-lagged associations* |  |  |  |  |
| Sleep fragment_(day 2)_ → Activity counts_(day 3)_ | -0.122 | -0.655 | 0.411 | .653 |
| Sleep fragment_(day 2)_ → Screen time_(day 3)_ | 0.007 | -0.011 | 0.025 | .442 |
| *(Day 2 → Day 3) Lagged association* |  |  |  |  |
| Sleep fragment_(day 2)_ → Sleep fragment_(day 3)_ | **0.208** | **0.053** | **0.363** | **.009** |
| Activity counts_(day 2)_ → Activity counts_(day 3)_ | **0.526** | **0.422** | **0.630** | **<.001** |
| Screen time_(day 2)_ → Screen time_(day 3)_ | **0.448** | **0.346** | **0.550** | **<.001** |
| *(Day 3) Cross-lagged associations* |  |  |  |  |
| Activity counts_(day 3)_ → Sleep fragment_(day 3)_ | 0.005 | -0.032 | 0.042 | .789 |
| Screen time_(day 3)_ → Sleep fragment_(day 3)_ | -0.093 | -0.981 | 0.795 | .838 |
| *Covariance^a^* |  |  |  |  |
| Activity counts_(day 1)_ ↔ Screen time_(day 1)_ | -5.862 | -14.094 | 2.370 | .163 |
| Activity counts_(day 2)_ ↔ Screen time_(day 2)_ | **-9.392** | **-16.436** | **-2.348** | **.006** |
| Activity counts_(day 3)_ ↔ Screen time_(day 3)_ | -5.926 | -12.284 | 0.432 | .060 |
| Screen time_(day 1)_ ↔ Screen time_(day 3)_ | **1.023** | **0.680** | **1.366** | **<.001** |
| Model data fit indices:  *x*^2^_(16)_ = 53.02(*P* <.001); RMSEA = .094 (.067, .122); CFI = .919; TLI = .823; SRMR = .050 | | | | |

b = unstandardized path coefficient; CI = confidence interval; RMSEA = root mean square error of approximation; CFI = comparative fit index; TLI = Tucker Lewis index

*Note.* Bold indicates statistically significant effects.

^a^ covariance between ‘activity counts_(day 1)_’ and ‘activity counts_(day 3)_’ was fixed to zero due to non-convergence of the model.
