# Supplementary material for: Examining the day-to-day bidirectional associations between physical activity, sedentary behavior, screen time, and sleep health during school days in adolescents
Source: PLoS One. 2020 Sep 3;15(9):e0238721. doi: 10.1371/journal.pone.0238721 (PMC7470331; doi:10.1371/journal.pone.0238721)
Supplement: S5 Table — (DOCX) [file pone.0238721.s005.docx]

**Supplement Table 5.**

**Autoregressive Cross-Lagged Path Model Analysis with Sleep Quality.**

| Temporality of association | b | 95% CI | | *P*-value |
| --- | --- | --- | --- | --- |
|  |  | Lower | Upper |  |
| *(Day 1) Cross-lagged associations* |  |  |  |  |
| Activity counts_(day 1)_ → Sleep quality_(day 1)_ | 0.0002 | -0.0003 | 0.0008 | .433 |
| Screen time_(day 1)_ → Sleep quality_(day 1)_ | -0.009 | -0.023 | 0.005 | .179 |
| *(Day 1 → Day 2) Cross-lagged associations* |  |  |  |  |
| Sleep quality_(day 1)_ → Activity counts_(day 2)_ | 8.454 | -18.400 | 35.308 | .537 |
| Sleep quality_(day 1)_ → Screen time_(day 2)_ | -0.853 | -1.900 | 0.194 | .110 |
| *(Day 1 → Day 2) Lagged association* |  |  |  |  |
| Sleep quality_(day 1)_ → Sleep quality_(day 2)_ | **0.680** | **0.568** | **0.792** | **<.001** |
| Activity counts_(day 1)_ → Activity counts_(day 2)_ | **0.518** | **0.406** | **0.630** | **<.001** |
| Screen time_(day 1)_ → Screen time_(day 2)_ | **0.482** | **0.376** | **0.588** | **<.001** |
| *(Day 2) Cross-lagged associations* |  |  |  |  |
| Activity counts_(day 2)_ → Sleep quality_(day 2)_ | -0.0002 | -0.0006 | 0.0003 | .514 |
| Screen time_(day 2)_ → Sleep quality_(day 2)_ | **-0.014** | **-0.026** | **-0.002** | **.021** |
| *(Day 2 → Day 3) Cross-lagged associations* |  |  |  |  |
| Sleep quality_(day 2)_ → Activity counts_(day 3)_ | 5.463 | 5.351 | 5.575 | .657 |
| Sleep quality_(day 2)_ → Screen time_(day 3)_ | 0.277 | -0.503 | 1.057 | .486 |
| *(Day 2 → Day 3) Lagged association* |  |  |  |  |
| Sleep quality_(day 2)_ → Sleep quality_(day 3)_ | **0.578** | **0.474** | **0.682** | **<.001** |
| Activity counts_(day 2)_ → Activity counts_(day 3)_ | **0.352** | **0.246** | **0.458** | **<.001** |
| Screen time_(day 2)_ → Screen time_(day 3)_ | **0.458** | **0.356** | **0.560** | **<.001** |
| *(Day 3) Cross-lagged associations* |  |  |  |  |
| Activity counts_(day 3)_ → Sleep quality_(day 3)_ | -0.0005 | -0.001 | 0.00003 | .066 |
| Screen time_(day 3)_ → Sleep quality_(day 3)_ | -0.007 | -0.019 | 0.005 | .278 |
| *Covariance^a^* |  |  |  |  |
| Activity counts_(day 1)_ ↔ Screen time_(day 1)_ | -5.862 | -14.094 | 2.370 | .163 |
| Activity counts_(day 2)_ ↔ Screen time_(day 2)_ | **-9.392** | **-16.436** | **-2.348** | **.006** |
| Activity counts_(day 3)_ ↔ Screen time_(day 3)_ | -5.926 | -12.284 | 0.432 | .060 |
| Screen time_(day 1)_ ↔ Screen time_(day 3)_ | **1.023** | **0.680** | **1.366** | **<.001** |
| Model data fit indices:  *x*^2^_(16)_ = 54.16 (*P* <.001); RMSEA = .099 (.072, .129); CFI = .942; TLI = .865; SRMR = .045 | | | | |

b = unstandardized path coefficient; CI = confidence interval; RMSEA = root mean square error of approximation; CFI = comparative fit index; TLI = Tucker Lewis index

*Note.* Bold indicates statistically significant effects.

^a^ covariance between ‘activity counts_(day 1)_’ and ‘activity counts_(day 3)_’ was fixed to zero due to non-convergence of the model.
